# Clinical Characteristics of Late‐Onset (≥ 65 Years) Ulcerative Colitis: A Single‐Center Retrospective Study

**DOI:** 10.1155/grp/8860301

**Published:** 2026-01-27

**Authors:** Ayumi Ito, Miki Koroku, Syun Murasugi, Maria Yonezawa, Teppei Oomori, Shinichi Nakamura, Katsutoshi Tokushige, Yousuke Nakai

**Affiliations:** ^1^ Department of Gastroenterology, Tokyo Women′s Medical University, Shinjuku-ku, Tokyo, Japan

**Keywords:** adverse effects, age-related change, late onset, ulcerative colitis

## Abstract

**Background:**

Late‐onset ulcerative colitis (UC) has become prevalent in recent years. The differences between late‐onset, middle aged–onset, and early‐onset UC have not been fully elucidated.

**Methods:**

The clinical characteristics, steroid side effects, remission rates, and surgical rates of UC patients were retrospectively studied. Patients were allocated to three groups according to age at diagnosis: late‐onset group (≥ 65 years), middle aged–onset (50–64 years), and early‐onset group (≤ 49 years).

**Results:**

Clinical characteristics such as admission age, duration of disease, history of cancer, days to diagnosis, clinical activity on admission, hemoglobin level on admission, and steroid use were significantly different between the three groups. The side effects of steroids were significantly more common in the late‐onset group. The remission induction rates differed significantly between the three groups: 67.7%, 71.7%, and 83.6% in the late‐onset, middle aged–onset, and early‐onset groups. Surgery rates were significantly higher in the late‐onset group: Surgery rates in the late‐onset, middle aged–onset, and early‐onset groups were 14.2%, 3.4%, and 3.4% at 12 months of diagnosis, and 23.8%, 10.3%, and 4.2% at 24 months of diagnosis.

**Conclusions:**

In conclusion, remission rates were low and surgical rates were higher with increasing age of onset. In addition, steroid‐related side effects increased with age. It was possible that late‐onset UC may have a different pathogenesis and be resistant to treatment.

## 1. Introduction

In recent years, the number of patients with ulcerative colitis (UC) has been increasing, particularly among older adults. [1] Aging is a global problem in developed countries including Japan, which has one of the highest super‐aged populations [2, 3]. Although age itself can affect the management of various diseases, including UC, its effects have not been fully elucidated, as elderly patients are often excluded from clinical trials due to comorbidities [4]. Although its onset age is often young, UC can occur at any age [5]. With an increasing old population, old‐onset of UC is also increasingly recognized, and there are some studies comparing older and younger UC [6]. However, it might be insufficient to simply consider only older and younger patients in this aging population. In particular, the risk of infection also increases with age, especially after the age of 50 years [7]. The aim of this retrospective study was to compare clinical characteristics and outcomes in the three groups by the onset age: late‐onset (65 years old and over), middle aged–onset (50–64 years), and early‐onset (49 years and younger).

## 2. Materials and Methods

Consecutive patients with UC who were hospitalized and treated at Tokyo Women′s Medical University Hospital, from April 2000 to January 2024, were retrospectively studied.

Patients were divided into three groups according to the age at onset: late‐onset group (onset at age 65 years or older), middle aged–onset group (onset at age 50–64 years), and early‐onset group (onset at age 49 years or younger). Patient characteristics, disease status, and treatment outcomes were compared between the groups. The early‐onset group included both adults and children, with onset observed as early as age 10.

The following data were extracted from the medical records: Sex, age at admission, age at disease onset; duration of disease; extent of disease (classified according to the Montreal classification: proctitis, left‐sided, and extensive); cancer history; days to diagnosis; Lichtiger clinical activity index (CAI) (used as a clinical activity index for UC, defined as remission if CAI is 4 or less); hemoglobin (Hb) level at admission; CRP levels; colonoscopy score (Mayo score); ulcerative colitis endoscopic index of severity (UCEIS); use of mesalazine; steroid dose during hospitalization, duration of hospitalization; induction remission therapy; steroid side effects; remission rate; surgery rates; and surgery rates within 12 and 24 months of diagnosis were examined [8–10].

Patient characteristics and steroid side effects were evaluated by per hospitalization analysis, not per patient analysis, but surgical rates within 12 and 24 months of diagnosis were evaluated by per patient analysis. The data used in this study were accessed on August 1, 2024 for research purposes.

### 2.1. Statistical Analysis

Data were presented as the number of cases or mean ± standard deviation (SD). The chi‐square test and Student′s *t*‐test were used for between‐group comparisons, and a *p* value of less than 0.05 was considered statistically significant. The results were evaluated with Kaplan–Meier curves and compared with logrank. Statistical analyses were performed with JMP Pro.15 (Statistical Discover from SAS).

### 2.2. Ethical Considerations

The study was approved by the Ethics Committee of Tokyo Women′s Medical University (Approval Number: 2024‐0006). Informed consent was obtained from all participants on an opt‐out basis given the retrospective nature of the study.

### 2.3. Ethics Statement

Oral informed consent was obtained from all participants and documented in their medical records. For minors, consent was obtained from their parents or legal guardians. For participants who had already completed their treatment and could not be contacted directly (due to end of outpatient treatment), an opt‐out approach was adopted to provide an opportunity to decline participation. Consent is detailed in the ethics statement in the Methods and online submission information.

## 3. Results

### 3.1. Patient Characteristics

A total of 359 cases (21 late‐onset, 58 in middle aged–onset, and 280 early‐onset) were identified and there were 614 hospital admissions: 31 in late‐onset group, 117 in middle aged–onset group, and 466 in early‐onset group.

In the per admission analysis, significant differences were observed among the three groups in the age at admission (73.3, 64.9, and 34.4 years old) and disease duration (4.1, 8.8, and 8.1 years) in the late, middle‐aged, and early‐onset groups. A history of cancer was also common and Hb level was low in the late‐onset group (Table 1). There were no significant differences in terms of site involved, CAI, colonoscopy scores (Mayo, UCEIS) on admission, as well as duration of hospitalization. Although there was no significant difference in the steroid dose during hospitalization, the rate of steroid use during remission induction therapy was lower in the late‐onset group: 83.8%, 91.4%, and 96.5% in the late‐onset, middle aged–onset, and early‐onset groups. On the other hand, there were no significant differences in other remission induction therapies.

**Table 1:** Patient characteristics per admission according to age at onset.

	**Late onset** **n** = 31	**Middle aged–onset** **n** = 117	**Early onset** **n** = 466	**p** **value**
Sex, male	12 (38.7)	72 (61.5)	277 (59.4)	0.11
Age at admission, years	73.3 ± 5.9	64.9 ± 8.0	34.4 ± 11.3	< 0.05
Duration of disease, years	4.1 ± 6.4	8.8 ± 8.8	8.1 ± 7.9	< 0.05
Site involved: proctitis/left‐sided colitis/extensive	0/24/7	1/95/21	4/107/355	0.53
History of cancer	4 (12.9)	15 (12.8)	2 (0.43)	< 0.05
Time to diagnosis, days	108.6 ± 229.5	81.1 ± 94.3	124.9 ± 443.1	< 0.05
Data on admission				
CAI	11.6 ± 3.2	12.0 ± 3.0	12.3 ± 3.2	0.31
Hemoglobin, g/dL	11.2 ± 1.7	11.9 ± 1.9	12.3 ± 2.3	< 0.05
CRP, mg/dL	4.4 ± 2.7	2.8 ± 4.3	3.5 ± 5.2	0.15
Mayo endoscopic score	2.8 ± 0.3	2.6 ± 0.6	2.6 ± 0.5	0.06
UCEIS score	6.3 ± 0.9	5.5 ± 1.5	5.7 ± 1.5	0.07
Use of mesalazine	26 (83.8)	107 (91.4)	440 (94.4)	0.06
Steroid dose during hospitalization, mg	559.4 ± 492.1	615.1 ± 453.9	787.2 ± 1206	0.68
Duration of hospitalization, days	31.0 ± 18.3	31.6 ± 12.1	31.7 ± 17.2	0.68
Remission induction therapy^a^				
PSL	26 (83.8)	107 (91.4)	450 (96.5)	< 0.05
CAP	12 (38.7)	36 (30.7)	120 (25.7)	0.41
Biologic	11 (35.4)	22 (22.6)	91 (19.5)	0.056
IFX/ADA/VED/UST/MIR	4/2/5/0/0	11/8/6/2/0	59/17/7/5/4/1	0.22
Tacrolimus	6 (19.3)	21 (17.9)	114 (24.4)	0.38
Cyclosporin	3 (9.6)	29 (24.7)	68 (14.5)	0.06
JAK inhibitor	0	1 (0.8)	3 (0.6)	0.44

*Note:* Data are shown in mean+/−SD or number (%).

Abbreviations: ADA, adalimumab; CAI, clinical activity index; CAP, cytapheresis; CRP, C‐reactive protein; IFX, infliximab; JAK inhibitor, janus kinase inhibitor; MIR, mirikizumab; PSL, predonisolone; UCEIS, ulcerative colitis endoscopic index of severity; UST, ustekinumab; VED, Vedolizumab.

^a^ ^a^Some patients received more than one treatment.

### 3.2. Side Effects of Steroids

Overall rates of steroid side effects were significantly higher in the late‐onset group (Table 2). Diabetes mellitus, osteoporosis, thrombosis, depression, cataract, and glaucoma were more often observed in the late‐onset group.

**Table 2:** Side effects of steroids according to age at onset.

	**Late onset** **n** = 26	**Middle aged–onset** **n** = 107	**Early onset** **n** = 450	**p** **value**
Any side effects	18 (69.2)	42 (39.2)	65 (14.4)	< 0.05
Moon‐like facies	3 (11.5)	4 (3.7)	23 (5.1)	0.27
Insomnia	3 (11.5)	5 (4.7)	10 (2.2)	< 0.05
Thrombosis	4 (15.3)	3 (2.8)	9 (2.0)	< 0.05
Depression	5 (19.2)	5 (4.7)	5 (1.1)	< 0.05
Osteoporosis	5 (19.2)	4 (3.7)	5 (1.1)	< 0.05
Fatty liver	1 (3.8)	3 (2.8)	4 (0.8)	0.16
Hypertension	1 (3.8)	4 (3.7)	3 (0.6)	0.16
Diabetes	1 (3.8)	3 (2.8)	2 (0.4)	< 0.05
Pneumocystis pneumonia	0	3 (2.8)	3 (0.7)	0.41
Steroid withdrawal syndrome	0	2 (1.8)	3 (0.7)	0.86
Glaucoma	2 (7.6)	1 (0.9)	4 (0.8)	< 0.05
Cataract	2 (7.6)	1 (0.9)	2 (0.4)	<0.05
Avascular necrosis of the femoral head	0	2 (1.8)	3 (0.7)	0.34
Steroid myopathy	0	0 (0)	2 (0.4)	0.74
Pancreatitis	0	0	2 (0.4)	0.74

*Note:* Numbers are shown in number (%). Some patients had more than one side effect.

### 3.3. Remission and Surgical Rates

**Figure 1:**
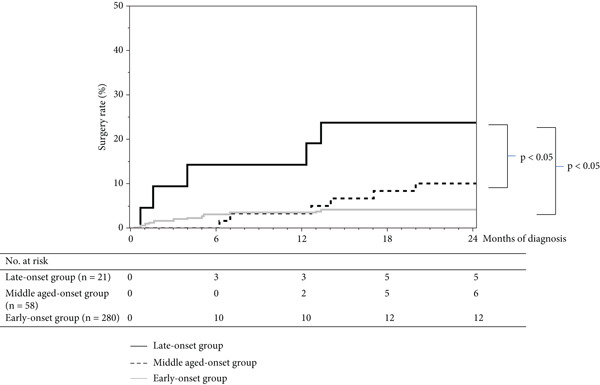
Surgery rate within 12 or 24 months of diagnosis.

The remission induction rates differed significantly between the three groups: 67.7%, 71.7%, and 83.6% in the late‐onset, middle aged–onset, and early‐onset groups. There were also significant differences in surgery rates at 12 and 24 months of diagnosis. Surgical rates were 14.2%, 3.4%, and 3.5% at 12 months and 23.8%, 10.3%, and 4.2% at 24 months in the late‐onset, middle aged–onset, and early‐onset groups, respectively (Figure 1 and Table 3).

**Table 3:** Remission and surgical rates according to age at onset.

	Late onset *n* = 31	Middle aged–onset *n* = 117	Early onset *n* = 466	*p* value

Remission	22 (67.7)	84 (71.7)	390 (83.6)	< 0.05

	Very late onset *n* = 21	Late onset *n* = 58	Early onset *n* = 280	*p* value

Surgery within 12 months of diagnosis	3 (14.2)	2 (3.4)	10 (3.5)	< 0.05
Surgery within 24 months of diagnosis	5 (23.8)	6 (10.3)	12 (4.2)	< 0.05

*Note:* Numbers are shown in *n* (%).

## 4. Discussion

Globally, aging populations are increasing, and life expectancy is rising. Furthermore, although UC can develop at any age, many studies divide their analysis into two groups: elderly and nonelderly. Therefore, this study divided participants into three age groups for analysis. This approach differs from previous reports [11].

In this single‐center retrospective study, three groups of UC patients with different ages at diagnosis were compared: late‐onset (onset at age 65 years or older), middle aged–onset (onset at age 50–64 years old), and early‐onset (onset at age 49 years or younger). The main findings were age at onset, duration of disease, history of cancer, days to diagnosis, clinical activity, Hb level on admission, and steroid use differed significantly between the groups. The side effects of steroids were significantly higher, especially in the late‐onset group. Furthermore, remission occurred less frequently in the late‐onset group, and surgery within 12–24 months of diagnosis was more common than in the other two groups.

The late‐onset group had a shorter time from onset to hospitalization, more cancer complications, and lower Hb levels. It is suggested that patients with late‐onset UC may require hospitalization earlier after onset than younger patients. It is also important to consider comorbidities, including preexisting cancer, in the elderly. Because aging increases the risk of developing all but a few cancers [12], and because the number of cancer patients is expected to increase as the population ages, treating physicians need to be aware of the relationship between cancer and UC treatment.

Low Hb levels are a complication of UC and are significantly associated with all‐cause mortality and morbidity, including cardiovascular disease, infections, physical disability, cognitive impairment, and increased hospitalizations. The risk of these complications has been reported to increase with age [13, 14].

In the present study, the time to diagnosis was shortest in the middle aged–onset group, longest in the early‐onset group, and similar in the late‐onset and early‐onset groups. This is different from previous reports. The reason for the difference is that in the previous report, nonyoung onset UC was considered less likely to be diagnosed due to the high prevalence of mild disease. In the present study, however, there was no difference in severity among the three groups. In addition, middle‐aged and older people are more likely than younger people to visit hospitals and undergo physical examinations for underlying diseases. Therefore, it is believed that they are being diagnosed earlier than in the past [15].

With regard to remission induction therapy, steroid use decreased with age; there is a large body of evidence supporting the efficacy of steroids in UC [16]. However, due to the increased risk of side effects such as infection and diabetes, steroids are often not used in older patients, making treatment of the late‐onset group difficult [17].

Side effects of steroids were present in all three groups (Table 2), but their prevalence increased with increasing age group, that is, was highest in the late‐onset group. The most common side effects in this age group were diabetes, osteoporosis, thrombosis, depression, cataracts, and glaucoma. In addition to steroid use, the effects of aging are likely to play a role in these side effects.

The use of nonsteroidal anti‐inflammatory drugs did not differ significantly between the three groups, but the late‐onset group had the lowest remission induction rate (Table 3). This finding is consistent with previous studies reporting lower remission rates in patients with late‐onset UC [18]. This suggests that the late‐onset group may be more resistant to treatment. This may be due to age‐related factors that reduce the effectiveness of drug therapy. Studies have shown that older people with Crohn′s disease have difficulty achieving effective drug therapy, including anti‐tumor necrosis factor drugs [19]. In addition, the use of immunosuppressive agents and biological therapies did not differ significantly among the three groups. Although safety considerations are necessary, such therapies are recommended in all groups when clinically indicated.

In recent years, immune function itself, in particular T cell function, has been reported to decrease with advancing age. When cells age, they acquire a senescence‐associated secretory phenotype (SASP) and consequently produce higher amounts of inflammatory cytokines and chemokines. SASP has been reported to be involved in chronic inflammatory diseases such as cardiovascular diseases and pneumonia [20, 21].

If SASP is also involved in UC, it is conceivable that it would make UC more treatment‐resistant with increasing age. Although many aspects of the pathology of UC remain unknown, immunity is believed to play a major role [22]. Future studies should investigate how changes in immune function with age affect the pathology of UC.

With regard to surgery rates, the surgery rate within 12–24 months after diagnosis was higher in the late‐onset group. The surgical rate within 12 months after diagnosis was similar to that in previous reports [23]. In many cases, the cycle of recurrence and remission occurs within 24 months after the onset of UC, but inflammation does not settle down until later [24]. Therefore, intensive treatment is often required in the first 24 months after disease onset. The fact that the late‐onset group is more likely to undergo surgery within 12–24 months of diagnosis than the other groups also suggests that this group is more refractory. Furthermore, postoperative complications make the treatment of the late group extremely difficult (Table 1and Figure 1).

The main limitations of this study are its retrospective nature, being a single‐center study, and the small sample size, particularly in the middle aged–onset and late‐onset groups. Further large multicenter studies are needed.

## 5. Limitations

The limitations of this study are that it is a retrospective study conducted at a single institution, so some degree of bias is to be expected. Another problem is the small number of elderly‐onset cases.

Future work includes increasing the number of cases and conducting a multicenter prospective study.

## 6. Conclusion

In conclusion, remission rates were low and surgical rates were higher with increasing age of onset. In addition, steroid‐related side effects increased with age. It was possible that late‐onset UC may have a different pathogenesis and be resistant to treatment. This study suggests that late‐onset UC is more refractory than other groups. Age‐related aspects should be fully considered when choosing a treatment for UC.

## Disclosure

An earlier version of this study was presented in abstract form at the ECCO Congress 2025. A.I., S.N., K.T., and N.K. approved the final version of the manuscript.

## Conflicts of Interest

The authors declare no conflicts of interest.

## Author Contributions

A.I. and K.T.: study concept and design; S.M., M.Y., T.O., and A.I.: data acquisition; A.I.: statistical analysis and drafting of the first version of the manuscript; A.I., S.N., K.T., and Y.N.: critical revision and approval of the final version of the manuscript.

## Funding

No funding was received for this manuscript.

## Data Availability

The data that support the findings of this study are available from the Clinical and Academic Research Promotion Center (CARP) of Tokyo Women′s Medical University. Access to the data is restricted due to ethical and privacy considerations but may be provided by the corresponding author upon reasonable request and with approval from the Ethics Committee.
